# Anti-Obesity Effect and Mechanism of Chitooligosaccharides Were Revealed Based on Lipidomics in Diet-Induced Obese Mice

**DOI:** 10.3390/molecules28145595

**Published:** 2023-07-23

**Authors:** Minchuan Zhou, Jingqing Huang, Jingwen Zhou, Cuiting Zhi, Yan Bai, Qishi Che, Hua Cao, Jiao Guo, Zhengquan Su

**Affiliations:** 1 Guangdong Engineering Research Center of Natural Products and New Drugs, Guangdong Provincial University Engineering Technology Research Center of Natural Products and Drugs, Guangdong Pharmaceutical University, Guangzhou 510006, China; zhouminchuan2021@163.com (M.Z.); jwzhou1018@163.com (J.Z.); 2 Guangdong Metabolic Disease Research Center of Integrated Chinese and Western Medicine, Key Laboratory of Glucolipid Metabolic Disorder, Ministry of Education of China, Institute of Chinese Medicine, Guangdong TCM Key Laboratory for Metabolic Diseases, Guangdong Pharmaceutical University, Guangzhou 510006, China; 19807730515@163.com (J.H.); mandyzhi@163.com (C.Z.); 3 Department of Pharmacy, Affiliated Hospital of Guilin Medical University, Guilin 541001, China; 4 School of Public Health, Guangdong Pharmaceutical University, Guangzhou 510310, China; angell_bai@163.com; 5 Guangzhou Rainhome Pharm & Tech Co., Ltd., Science City, Guangzhou 510663, China; cheqishi@rhkj.com.cn; 6 School of Chemistry and Chemical Engineering, Guangdong Pharmaceutical University, Zhongshan 528458, China; caohua@gdpu.edu.cn

**Keywords:** AMP-activated protein kinase, chitooligosaccharides, lipidomics, obesity

## Abstract

Chitooligosaccharide (COS) is a natural product from the ocean, and while many studies have reported its important role in metabolic diseases, no study has systematically elaborated the anti-obesity effect and mechanism of COS. Herein, COSM (MW ≤ 3000 Da) was administered to diet-induced obese mice by oral gavage once daily for eight weeks. The results show that COSM administration reduced body weight; slowed weight gain; reduced serum Glu, insulin, NEFA, TC, TG, and LDL-C levels; increased serum HSL and HDL-C levels; improved inflammation; and reduced lipid droplet size in adipose tissue. Further lipidomic analysis of adipose tissue revealed that 31 lipid species are considered to be underlying lipid biomarkers in COS therapy. These lipids are mainly enriched in pathways involving insulin resistance, thermogenesis, cholesterol metabolism, glyceride metabolism and cyclic adenosine monophosphate (cAMP), which sheds light on the weight loss mechanism of COS. The Western blot assay demonstrated that COSM intervention can improve insulin resistance, inhibit de novo synthesis, and promote thermogenesis and β-oxidation in mitochondria by the AMPK pathway, thereby alleviating high-fat diet-induced obesity. In short, our study can provide a more comprehensive direction for the application of COS in obesity based on molecular markers.

## 1. Introduction

Obesity has become an important global health problem [1]. According to the report of experts attending the 2022 World Obesity Day China Summit Forum states, more than 40% (2 billion) of adults worldwide are overweight or obese. Obesity is defined as abnormal or excessive fat accumulation in the body that may impair health and is closely associated with multiorgan and multisystem diseases, such as cardiovascular diseases, diabetes mellitus, liver disease, dyslipidaemia, osteoarthritis, and kidney failure [2,3,4]. Moreover, the New England Magazine survey found that obesity is linked to 13 types of cancers [5]. At present, obesity is mainly treated through diet control and exercise, but this is hard to achieve in the modern fast-paced lifestyle. Thus, it is necessary to find more treatment targets and develop safer and more effective drugs for obesity.

The most notable features of obesity are the lipid metabolism disorders; however, the composition of lipids is very complex [6,7]. Conventional methods of lipid determination cannot achieve the pathophysiological description of obesity [8]. Therefore, high-resolution and high-sensitivity analysis techniques are required to elucidate the relationship between differential lipids and obesity. Lipidomics analysis is a powerful method for detecting and monitoring fine-grained changes in various lipid species at the molecular level [9]. For example, the lipidomics approach has demonstrated that polysaccharides isolated from fermented M. charantia juice changed 21 lipids in the serum of obese rats [10]. Moreover, broad targeted lipidomics that integrates the advantages of non-targeted and targeted metabolic detection technology can achieve high-throughput, high-sensitivity, and wide-coverage targeted metabolic lipid detection [8,9]. Thus, in this study, broad targeted lipidomics (uniformly called “lipidomics”) research methods based on UPLG-Q-TOF-MS instrumental analysis were used to identify obesity-related differences in heterometabolic lipids.

Natural products have attracted widespread attention in the field of biomedicine. Chitosan (CTS) is a natural polymer polysaccharide that is abundant in the carapace of marine arthropods, such as shrimp and crabs, and is extremely widely distributed. Chitooligosaccharides or chitosan oligosaccharides (COS) are produced by enzymatic hydrolysis or acidic hydrolysis of chitosan, which is a short-chain, low-molecular-weight functional oligosaccharide [11]. In vivo and in vitro studies have shown that chitooligosaccharides have higher solubility, lower viscosity, and higher absorption than chitosan [12,13,14]. COS has attracted much research interest in the field of functional food and biomedicine due to its various beneficial bioactivities, such as antioxidant, anti-inflammatory, antibacterial, antitumour, and antimetabolic disorder activities [12,15,16,17,18]. Essentially, COS is characterized by a degree of deacetylation (DD) of >90%, a degree of polymerization (DP) of <20, and an average molecular weight (MW) of <3900 Da [19,20]. However, the definition of COS is not restricted to these specifications. Abundant studies have utilized COS of varying MW, DD, and DP to verify their theoretical hypothesis. Although COS has been widely reported as a functional anti-obesity food, the precise biochemical mechanism of COS needs to be fully studied.

To date, no studies have applied lipidomics to investigate the efficacy of COS and clarify the potential mechanism of COS in regulating lipid biomarkers. In past experiments, Jin et al. [21] found that COS with a molecular weight not exceeding 3000 Da (COSM) has a higher binding capacity for fat and cholesterol through electrostatic adsorption than ordinary COS, which may be combined with dietary fat in the digestive tract and can then be excreted in the faeces. Based on the above research, we used chitooligosaccharides with molecular weights less than 3000 Da as the study material. Thirty-two male C57BL/6J mice with high-fat diet (HFD)-induced obesity were used to investigate the effect of COSM on lipid accumulation by measuring serum lipid levels. Orlistat was used as a positive control. Biochemical analysis and lipidomics were used to preliminarily explore the relevant mechanisms. The findings of this study might provide a theoretical basis for the development and application of COSM in functional foods.

## 2. Results

### 2.1. COSM Reduced Diet-Induced Weight Gain and Fat Pad Weight

**Figure 1:**
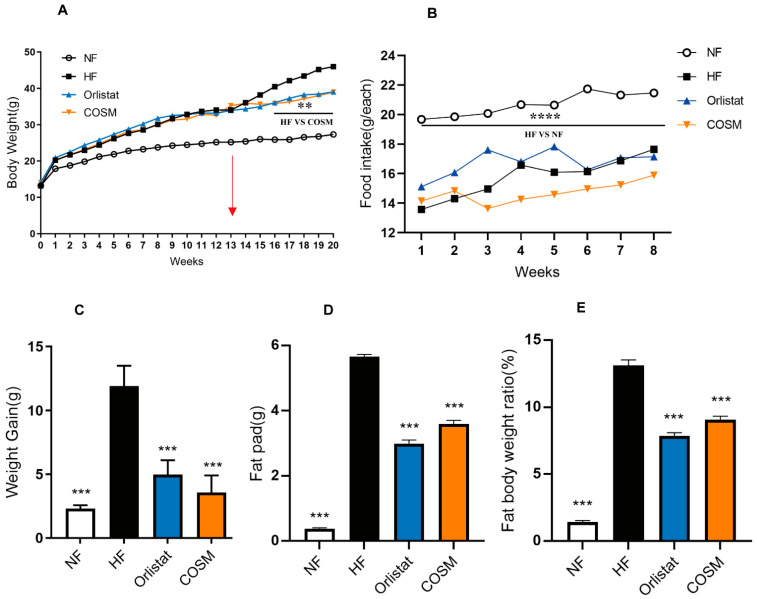
Effect of COSM supplementation on weight loss. (**A**) The BWs of mice in the four groups. (**B**) Food intake of mice in the four groups during administration. (**C**) Weight gain of mice in the four groups during the dosing period. (**D**) Fat pads of mice in the four groups during the dosing period. (**E**) Fat-to-body weight ratio of mice in the four groups during the dosing period. Note: The data are expressed as the mean ± SEM; n = 8. Compared with the HF group, ** *p* < 0.01, *** *p* < 0.001 and **** *p* < 0.0001.

To confirm whether COSM exhibits anti-obesity effects, we used HFD-fed C57BL/6J mice as an obesity model; mice were fed the HFD for 12 consecutive weeks. Then, the successfully modelled mice were administered the corresponding drug daily for 8 consecutive weeks. At the start of the experiment, there was no significant difference in body weight among the groups. After 12 weeks of feeding a high-fat diet, the average body weight of the mice fed an HFD reached more than 32 g and was higher than that of mice in the NF group (*p* < 0.01). The obesity index was greater than 20%, which indicated that the nutritionally obese mice were successfully modelled (Table 1). Compared with the NF group, the groups fed an HFD gained more weight rapidly during the modelling period (Figure 1A).

As shown in Table 1 and Figure 1A, during administration, the average body weights of the COSM and orlistat groups were markedly lower than that of the HF group (*p* < 0.01). The trend of weight gain in the COSM group was similar to that in the orlistat group (Figure 1A). Moreover, the average food intake of the mice in the NF group was higher than the food intake of the mice fed the HFD, which verified that the HFD had an effect on the appetite of the mice. However, there was no significant difference among the COSM group, orlistat group, and HF group, indicating that COSM did not significantly affect the appetite of mice (Figure 1B).

At the end of administration, compared with that in the HF group, the body weight gain in the COSM and orlistat groups had decreased significantly (Figure 1C). Moreover, COSM reduced the fat pad and fat body ratio in obese mice (Figure 1D,E). In conclusion, COSM can reduce weight without affecting appetite. Compared to weight loss drugs that suppress appetite, we considered COSM to be a safe and effective weight loss drug. However, its anti-obesity mechanism requires more intensive research.

### 2.2. COSM Regulated Glycolipid Metabolism Homeostasis

#### 2.2.1. Effect of COSM Supplementation on Glycometabolism in Obese Mice

**Figure 2:**
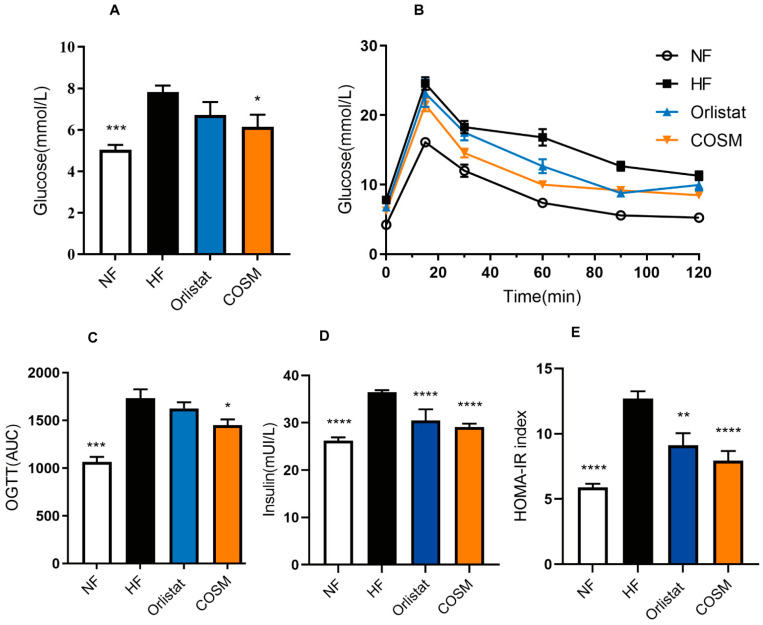
Effect of COSM supplementation on glycometabolism in obese mice. (**A**) Fasting blood glucose. (**B**,**C**) Oral glucose tolerance. (**D**) Serum insulin levels. (**E**) HOMA-IR. Note: The data are expressed as the mean ± SEM; n = 6–8. Compared with the HF group, * *p* < 0.05, ** *p* < 0.01, *** *p* < 0.001, and **** *p* < 0.0001.

Obesity induced by an HFD is often closely related to abnormal glucose and lipid metabolism. Carbohydrates are mainly absorbed in the gastrointestinal tract in the form of glucose and are directly used by red blood cells as the main energy source. However, in fat cells, excess glucose is metabolized into fatty acids and glycerol-3-phosphate, which combine to produce TGs [22]. As shown in Figure 2A, compared with that of the mice in the NF group, the fasting blood glucose (FBG) of mice with HFD-induced obesity was significantly increased and exceeded the normal blood glucose range [23]. This indicates a significant decrease in glucose tolerance in HFD-fed mice. Compared with that of the mice from the HF group, the blood glucose level of the mice treated with COSM decreased significantly (*p* < 0.05). Glucose tolerance was improved after the administration of COSM treatment. In addition, Figure 2B,C showed that the blood glucose at 0 min, 15 min, 30 min, 60 min, 90 min, and 120 min after fasting and oral glucose administration was higher in obese HFD-fed mice than in NF group mice. Additionally, the AUC for the oral glucose tolerance test (OGTT) was higher for HFD-fed mice than for the NF group mice. Compared with the HF group, the COSM group demonstrated a significant reduction in the abnormal increase in blood glucose caused by the HFD (*p* < 0.05). Importantly, high concentrations of plasma insulin are considered a biomarker of hepatic insulin resistance (IR). In the case of IR, it is necessary to increase insulin levels to metabolize glucose and inhibit glucose production in the liver. The levels of plasma insulin were notably decreased after COSM administration (Figure 2D). This result indicated an improvement in IR. We further calculated the HOMA-IR index by fasting glucose and insulin levels and found that HOMA-IR was significantly reduced in the COSM group compared with the HF group (Figure 2E). These results suggest that COSM effectively improved glycometabolism in obese mice.

#### 2.2.2. Effect of COSM Supplementation on Lipid Metabolism in Obese Mice

**Figure 3:**
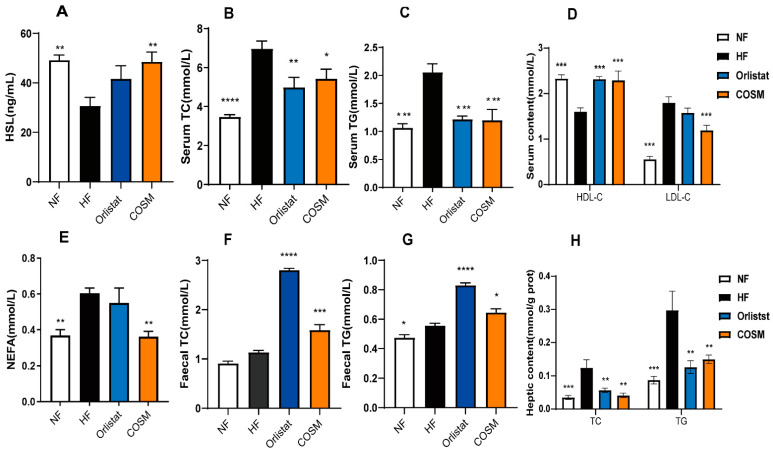
Effect of COSM supplementation on lipid metabolism in obese mice. (**A**) Serum HSL levels. (**B**–**D**) Levels of four blood lipids, namely, TC, TG, LDL-C, and HDL-C. (**E**) Serum NEFA levels. (**F**) Faecal TC levels. (**G**) Faecal TG levels. (**H**) Levels of liver TC and TG. Note: The data are expressed as the mean ± SEM; n = 6–8. Compared with the HF group, * *p* < 0.05, ** *p* < 0.01, *** *p* < 0.001, and **** *p* < 0.0001.

HSL was the first lipolytic enzyme discovered to breakdown triglycerides, and it is crucial in the process of lipolysis. Compared with those of the NF group, the serum HSL levels of the HF group were significantly decreased, but these levels were increased after COSM treatment. These findings indicate that COSM can regulate serum HSL homeostasis, thus promoting adipocyte hydrolysis (Figure 3A). Circulating NEFA has a high sensitivity to metabolic disorders in obesity. The HFD induced a distinct increase in NEFA levels in obese mice. Compared with that in the HF group, the level of NEFAs was significantly decreased in the COSM group. However, orlistat failed to reduce the level of NEFAs in the circulation (Figure 3E). Moreover, the serum levels of TC, TG and LDL-C in the mice in the HF group were all significantly increased, whereas the level of HDL-C was significantly decreased. COSM significantly reduced the TG, TC, and LDL-C levels in mice with HFD-induced obesity. Compared with that in the HF group, the level of HDL-C was elevated in the COSM group. In the orlistat group, only the TG level and TC level were reduced, and the level of HDL-C was increased (Figure 3B–D). Furthermore, we also measured the contents of faecal TC and faecal TG. There were no significant differences in faecal TC and faecal TG between the NF group and HF group. However, compared with those in the HF group, the contents of faecal TC and faecal TG in the COSM group and orlistat group were significantly increased (Figure 3F,G). Hence, we think COSM could promote lipid excretion from the digestive tract to achieve some anti-obesity effect. The liver is the main organ that regulates lipid metabolism and is closely related to TC biosynthesis and TG oxidative decomposition. TG and TC levels were significantly increased in the liver tissue of the HF group. The liver TG and liver TC levels in the COSM group and NF group were lower than those in the HF group (Figure 3H). In summary, COSM can alleviate glucose and lipid metabolism disorders caused by obesity.

### 2.3. COSM Suppressed Adipocyte Hypertrophy and Inflammation in Adipose Tissue

**Figure 4:**
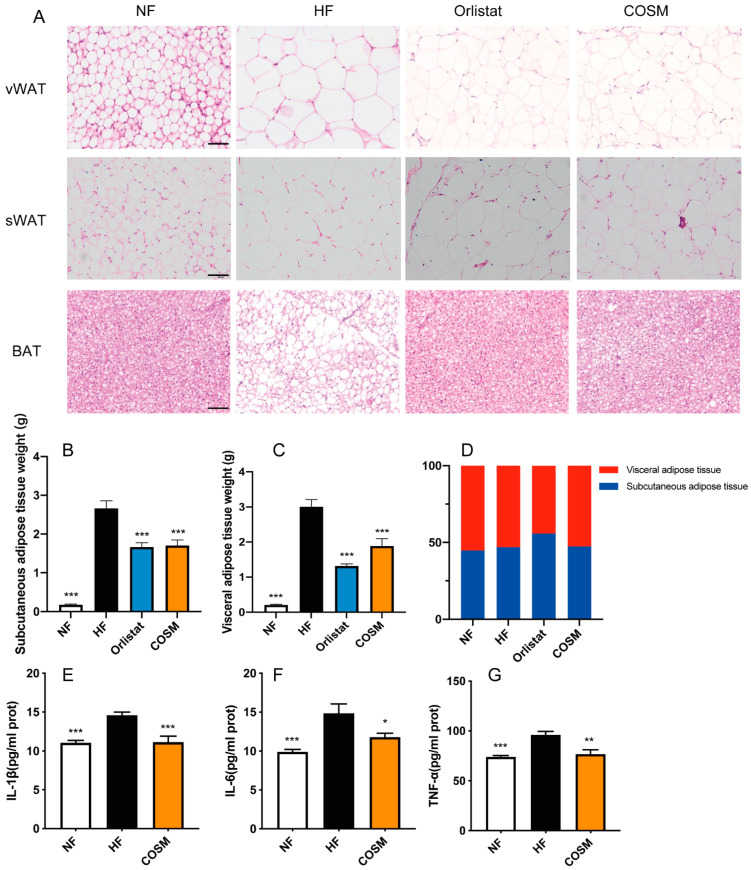
Effect of COSM supplementation on adipose tissue in obese mice. (**A**) Three adipose tissue pathological sections of mice in the four groups. Scale bar, 50 μm. (**B**) Mass of subcutaneous adipose tissue. (**C**) Mass of visceral adipose tissue. (**D**) The proportion of subcutaneous adipose tissue and visceral adipose tissue in mice in the four groups. (**E**–**G**) Associated inflammatory factors in visceral adipose tissue. Note: The data are expressed as the mean ± SEM; n = 8. Compared with the HF group, * *p* < 0.05, ** *p* < 0.01, and *** *p* < 0.001.

As fat storage increases, the size of fat cells increases. Pathological analysis via H&E staining showed that adipocytes in the vWAT and sWAT in the HF group were not uniform in size but were generally large and had fat vacuoles with large inner diameters. In contrast, the adipocytes in the NF group were arranged neatly and tightly, and the inner diameter of the vacuoles was smaller. Compared with the HF group, the COSM group demonstrated a reduction in fat cell size in the vWAT and sWAT. BAT in the scapular region was filled with many lipid droplets. The inner diameter of cells was significantly increased, and the cell membrane was thinner in the HF group than in the NF group. Due to excessive lipid droplets in the cytoplasm, the cells were not arranged neatly. Conversely, the BAT adipocytes in the COSM group were significantly smaller and more uniform in size, which more closely matched observations in the NF group (Figure 4A).

Obesity is related to the distribution of white fat. As depicted in Figure 4B,C, mice in the HF group had the greatest sWAT and vBAT fat weight. COSM improved the total fat in obese mice, and the total fat mass was smaller in the COSM group than in the HF group. In the orlistat group, the proportion of sWAT was larger than that of vWAT, but in the other three groups, the proportion of white fat was relatively average (Figure 4D). Through evaluation of the tissue structure, the fat cells in vWAT were found to be larger than those in sWAT (Figure 4A). This relates to the difference in cell structure in different adipose tissues. Obesity is generally characterized by low-grade chronic inflammation in adipose tissue, which is common in visceral fat. Compared with sWAT, vWAT has more inflammatory cells, and the fat cells are larger [24]. Compared with the HF group, the COSM group showed reduced levels of TNF-α, IL-1β, and IL-6 (Figure 4E–G). These results indicated that COSM intervention can improve the cell size in the three types of adipose tissues and improve the levels of inflammatory factors in adipose tissue.

### 2.4. Screening for Biomarkers of Subcutaneous Fat Metabolism

**Figure 5:**
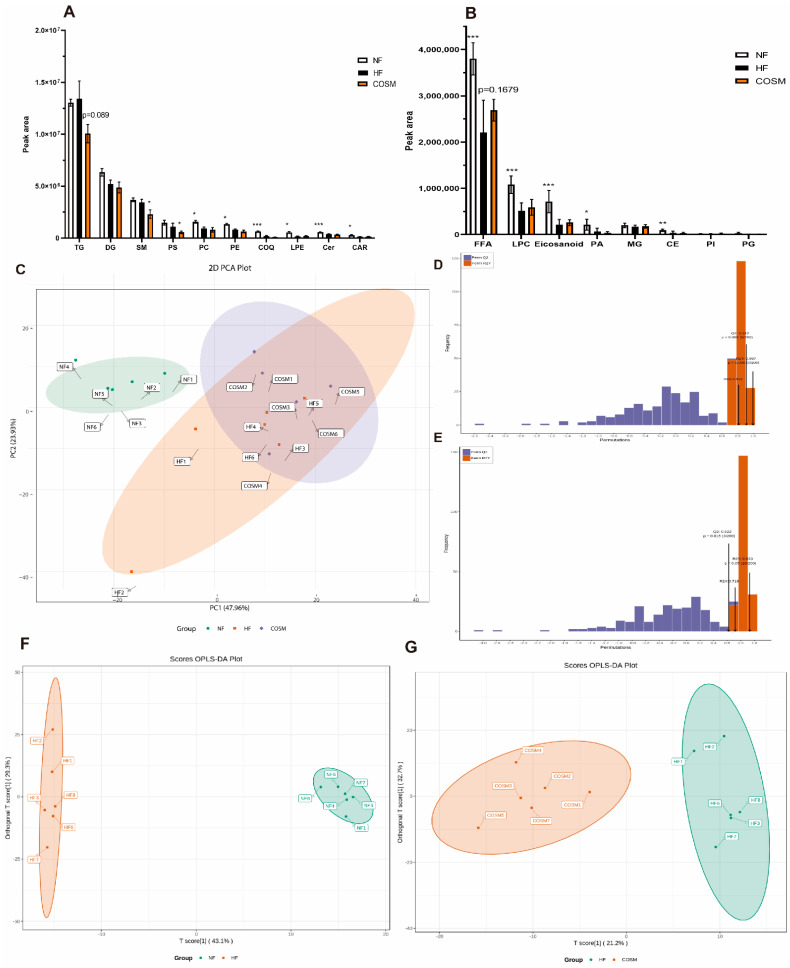
General analysis of subcutaneous adipose tissue lipidomics profiles in obese mice. (**A**,**B**) Various lipid levels in the three groups of mice. (**C**) PCA plots of the three groups of mice. (**D**) Permutation verification chart of the HF and NF groups. (**E**) Permutation verification chart of the COSM group vs. HF group. (**F**) OPLS-DA plots of the HF and NF groups. (**G**) OPLS-DA plot of the COSM group vs. HF group. Note: The data are expressed as the mean ± SEM; n = 6. Compared with the HF group, * *p* < 0.05, ** *p* < 0.01, and *** *p* < 0.001.

To further study whether lipid biomarkers are related to the biological activity of COSM, we analysed the different lipid compositions among the NF group, HF group and COSM group by lipidomics. A total of 598 lipid metabolites from 17 major lipid classes were identified in the subcutaneous adipose tissue; these included 250 TGs, 76 phosphatidylcholines (PCs), 68 phosphatidylethanolamines (PEs), 59 diglycerides (DGs), 32 FFAs, 24 ceramides (Cers), 23 sphingomyelins (SMs), 20 lysophosphatidylcholines (LPCs), 13 lysophosphatidylethanolamines (LPEs), 11 carnitines (CARs), 8 eicosanoids, 4 phosphatidic acids (PAs), 3 cholesterol esters (CEs), 2 monoglycerides (MGs), 2 phosphatidyl inositols (PIs), 1 phosphatidylserine (PS), 1 phosphatidylglycerol (PG), and 1 coenzyme Q (COQ). Figure 5A,B showed that the TG (*p* = 0.089) and DG contents were lower, and the FFA content was higher in the COSM group than in the HF group (*p* = 0.1679). SMs and PS were significantly different between the HF group and COSM group. The total content of other lipid metabolites was not significantly different between the HF group and COSM group.

To further compare and examine these groups, PCA was conducted to investigate the differences between groups. Figure 5C showed a significant difference between the HF group and the NF group. The HF group and the COSM group showed aggregation and failed to show proper intergroup separation, but one member of the HF group was delocated. Compared with PCA, OPLS-DA can maximize the distinction between groups, which is conducive to finding different metabolites. Subsequently, we obtained two multivariate OPLS-DA statistical models, and the NF and HF groups showed good adaptability (R2X = 0.813, R2Y = 0.997) and predictability (Q2 = 0.912) (Figure 5D). The HF group and COSM group model also showed good adaptability (R2X = 0.718, R2Y = 0.933) and predictability (Q2 = 0.622) (Figure 5E). These results show that the OPLS-DA models can effectively distinguish among the different groups and can be used as predictive models to effectively evaluate changes in the lipidomics profile (Figure 5F,G).

**Figure 6:**
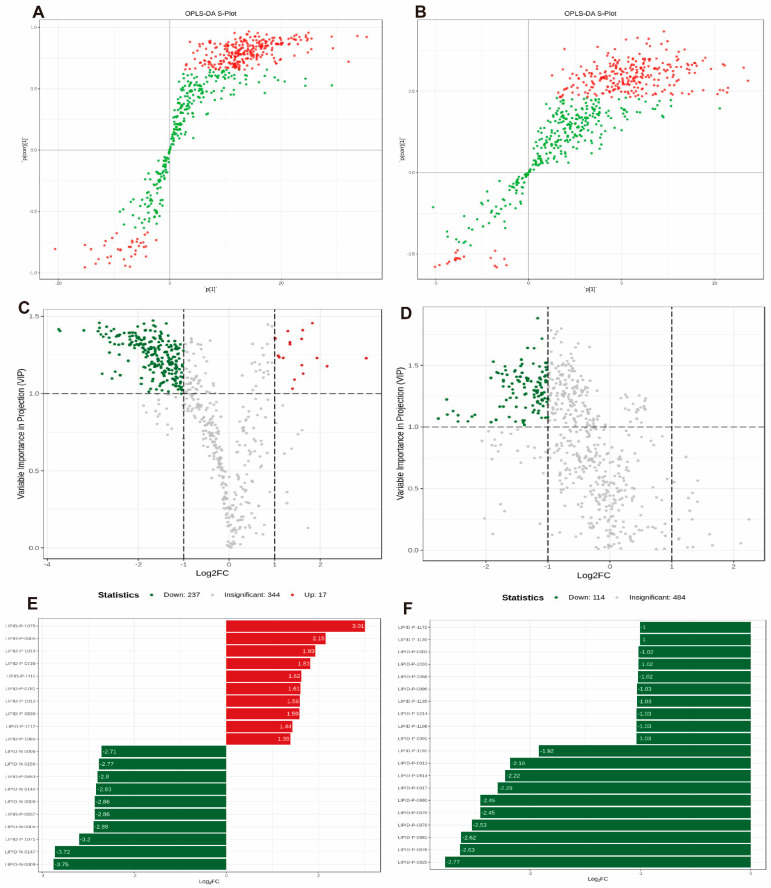
Differential metabolite screening for subcutaneous fat tissue in obese mice. (**A**) OPLS-DA S-plot of the HF vs. NF groups. (**B**) OPLS-DA S-plot of the COSM group vs. HF group. (**C**,**D**) Volcano plot analysis of the NF vs. HF and HF vs. COSM groups. (**E**) Difference multiples bar chart analysis of the NF vs. HF groups. (**F**) Difference multiples bar chart analysis of the HF vs. COSM groups. Note: The data are expressed as the mean ± SEM; n = 6.

A combination of the S-plot with variable importance in projection (VIP) > 1, Fold Change (FC) ≥ 2, and T test (*p* < 0.05) was used to screen potential biomarkers. The red dots in Figure 6A,B indicated that the VIP value of those metabolites is greater than 1, and the green dots indicated that the VIP value of the metabolites is less than 1. A total of 254 different lipid metabolites from 14 major lipid classes were identified in the subcutaneous adipose tissue from the NF group and the HF group. Of these, 237 metabolites were downregulated, and 17 metabolites were upregulated (Figure 6C). The classification revealed that these metabolites consisted of 65 TGs, 41 PCs, 41 PEs, 24 FFAs, 23 DGs, 17 LPCs, 12 LPEs, 11 Cers, 7 eicosanoids, 7 SMs, 2 PAs, 2 CARs, 1 COQ, and 1 CE (Supporting Information Figure S1. In addition, 114 differential metabolites from 5 major lipid classes, including 106 TGs, 4 DGs, 2 PEs, 1 Cer, and 1 SM, were identified between the HF group and COSM group, and they were markedly downregulated (Figure 6D, Supporting Information Figure S2). After the relative quantitative and qualitative analysis of the detected metabolites, we compared the difference multiples of the quantitative information between the NF group and the HF group, the HF group, and COSM group, respectively. The top 20 metabolites with the most significant differences are shown in Figure 6E,F. Figure 6E showed the HF group was upregulated by 9 TGs and 1 SM; the NF group was upregulated by 3 arachidonic acids, 3 LEPs, 2 PEs, 1 TG, and 1 FFA (corresponding database index and marker). Difference multiples bar chart analysis vindicated that the TGs content of the HF group were significantly upregulated, and the droplet diversity was reduced compared with the NF group. Moreover, Figure 6F shows the HF group was upregulated by 18 TGs and 2 DGs compared with the COSM group, which is consistent with Figure 5A,B. These results showed that COSM improves lipid metabolism in nutritionally obese mice by altering the abundance of TG, DG, PE, and other lipid metabolites. The differentiators excavated by lipidomics laid a theoretical foundation for comprehensive research on COSM.

### 2.5. Exploration of the Mechanism by which COSM Treatment Improves Lipid Metabolism in sWAT

**Figure 7:**
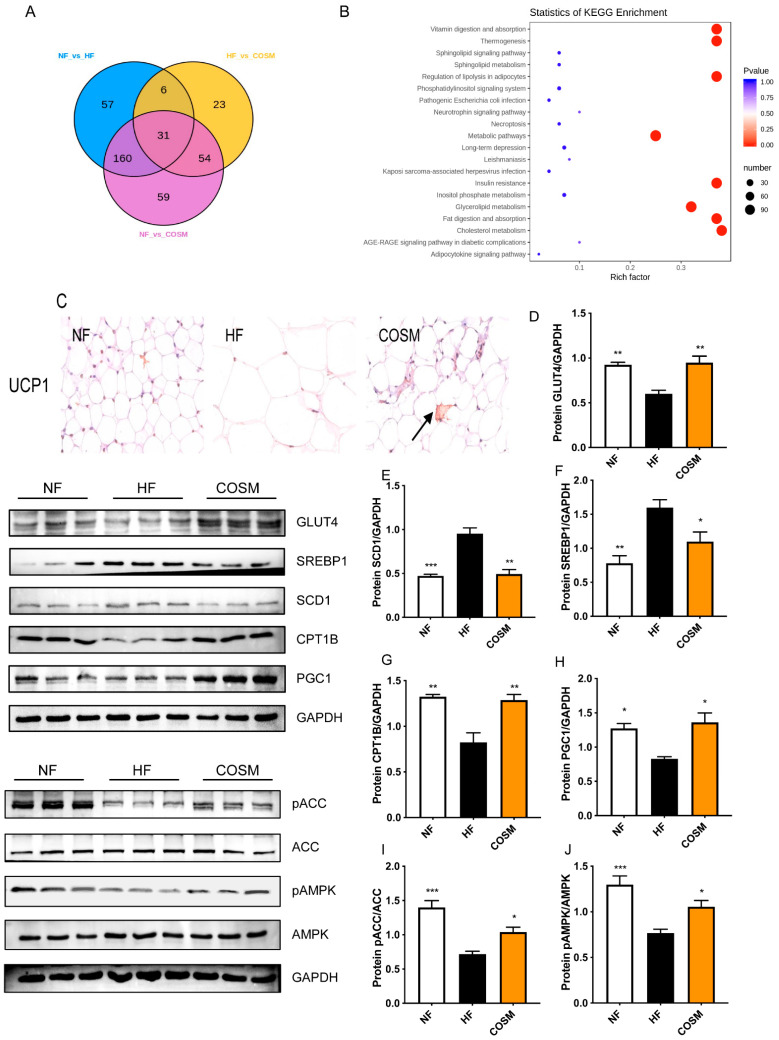
The potential mechanism of COSM in the treatment of nutritionally obese mice. (**A**) Venn diagram of the three groups of mice. (**B**) KEGG enrichment map. (**C**) sWAT immunohistochemical determination of UCP1 expression. The arrow points to UCP1 protein precipitation (200×). (**D**–**J**) The effect of COSM on fatty acid metabolism, cholesterol metabolism, and thermogenesis pathway-related protein expression in the subcutaneous fat of nutritionally obese mice (n = 3, mean ± SEM). Note: Compared with the HF group, * *p* < 0.05, ** *p* < 0.01, and *** *p* < 0.001.

According to the results described in Section 2.4, the differential markers between the groups were screened with a Venn diagram to identify common metabolic biomarkers. The relationships between the different metabolites in each group were assessed, and the differential metabolites were subjected to KEGG pathway enrichment analysis (KEGG database, https://www.genome.jp/kegg/pathway.html (accessed on 2 April 2019)). Figure 7A shows that 31 different metabolites were obtained from the analysis of the NF group, HF group, and COSM group. These differentially enriched substances, including 27 TGs, 2 PEs, 1 DG, and 1 SM, are mainly related to cholesterol metabolism, glycerolipid metabolism, glycerophospholipid metabolism, sphingolipid metabolism, insulin resistance, fat digestion and absorption, thermogenesis, the cAMP signalling pathway, and the sphingolipid signalling pathway (Figure 7B, Table 2).

UCP1 acts as a master regulator of thermogenesis in adipose tissue. COSM increased the expression of UCP1 in subcutaneous adipose tissue; this finding indicated that COSM can promote subcutaneous fat thermogenesis (Figure 7C). Obesity is highly correlated with glyceride metabolism and cholesterol metabolism. GLUT4, CPT1B, and PGC1 protein expression in the HF group mice was markedly lower than that in the NF group. Additionally, the cell transport of glucose was reduced, and acyl-CoA transport to mitochondria for fatty acid oxidation was blocked. COSM treatment significantly increased the expression of GLUT4, CPT1B, and PGC1 (Figure 7D,G,H). However, in the HF group, SCD1 and SREBP1 protein expression was upregulated, and endogenous fatty acid synthesis in fat cells was accelerated. The expression of SCD1 and SREBP1 in COSM-treated mice was markedly downregulated (Figure 7E,F). These results show that COSM administration can significantly improve glucose metabolism and mitochondrial lipid transport in adipocytes and inhibit de novo fatty acid synthesis. AMPK plays an important role in the regulation of fat metabolism. As shown in Figure 7I,J, pAMPK and pACC protein expression was reduced in the HF group, and pAMPK and pACC protein levels were significantly upregulated after COSM administration, indicating that AMPK pathway-mediated regulation of glyceride metabolism and cholesterol metabolism may be the mechanism of weight loss.

## 3. Discussion

In the present study, we generated a nutritionally obese mouse model by feeding the mice a high-fat diet. The successful index of the model mice included body weight, weight gain and the calculated obesity index. According to the above experimental data, the obese mouse model was successfully achieved. Then, we treated mice with COSM to examine its anti-obesity effect, and the daily physiological state of the mice was good after administration. The weight and weight gain of the mice treated with COSM were significantly lower than those of the mice that were not treated with COSM. During dosing treatment, mice in the COSM group had slightly lower food intake than mice in the HF group, but the difference was not significant. Thus, we think the main reason for weight loss is does not relate to changes in appetite; it may relate to breakdown and metabolism. Next, we collected the faeces of mice for TC and TG content detection and found that the TC and TG contents in the faeces of the COSM-treated mice were significantly increased. This may partly explain why there was no significant difference in food intake but a change in weight loss. After completion of the experimental period, the fat pads of mice were weighed, and the fat weight and fat-to-body ratio were lower in the COSM group than in the HF group. Considering the close relationship between glycolipid metabolism and obesity, we measured FBG, fasting insulin, OGTT, and HOMA-IR index values. The results showed that feeding an HFD can lead to disorders of glucose metabolism and the occurrence of insulin resistance (IR). Treatment with COSM can improve insulin resistance and regulate disorders of glucose metabolism. In addition, HSL is strictly controlled by insulin and plays a key gating role in the biological breakdown of TG. The inhibitory effect of insulin on HSL is associated with multiple metabolic pathways. FFAs can also interact with the insulin signalling pathway to promote the development of IR [25]. Therefore, we measured the levels of serum HSL and NEFA. The results showed that a high-fat diet caused insulin resistance and further accelerated the occurrence of lipid metabolism disorders. However, COSM improved insulin resistance and lipid metabolism disorders. Moreover, the levels of TC, TG, LDL-C, and HDL-C were closely related to dyslipidaemia. In addition, IR plays a key role in the lipid hydrolysis of adipose tissue, which can induce the transport of excess FFAs and accelerate the development of lipotoxicity [26]. Our results showed that liver TC and liver TG were significantly decreased in the COSM group, indicating that COSM was able to improve the occurrence of lipotoxicity.

Obesity is defined as excessive lipid accumulation and includes the hypertrophy and hyperplasia of adipocytes [27]. We performed pathological section analysis of sWAT, vWAT, and BAT. COSM treatment significantly reduced the size of lipid droplets. Moreover, considering that chronic inflammation is associated with obesity and can further worsen IR, we measured the levels of TNF-α, IL-1β, and IL-6 in visceral adipose tissue [28]. The level of inflammation increased after HFD feeding, while COSM improved inflammation to some extent. Dietary lipids are absorbed by adipocytes and esterified into TG. They are stored in the form of lipid droplets (LDs) in WAT. WAT is a key factor in controlling the energy balance of the whole body. It has a unique TG storage function that fuels other organs during periods of low carbohydrate utilization [29]. However, LDs in adipose tissue contain a variety of components, and the specific composition of the LDs is not clear [30]. Lipidomics analysis was carried out by us to explore ingredient differences among the NF group, HF group, and COSM group. Lipidomics revealed that the lipid profile of mice with HFD-induced obesity is characterized by elevated TG levels, while COSM treatment of obese mice led to a significant decrease in TG levels. This result also confirmed the increased serum TG and TC levels reported in obese mice [31,32]. Adipose TG lipase (ATGL) converts TG to DG. Then, DG is hydrolysed into MG by HSL, and monoglyceride lipase (MGL) hydrolyses MG into glycerol and FFA. Only adipose tissue can transport FFAs into the circulatory system [25,33]. Through lipid analysis, COSM was found to decrease the levels of TG, DG, and MG in sWAT and to increase the level of FFAs. Moreover, COSM was shown to regulate lipid metabolism in subcutaneous fat through a lipolytic pathway. DG (16:1/20:4/0:0), DG (18:3/18:3/18:3), DG (14:0/20:5/22:4), DG (18:2/18:3/20:4), and DG (14:0/20:3/22:6) were found to be markers of glycerolipid metabolism, which may closely relate to the TG lipolysis process. PE promotes fat production, increasing the content of lipid droplets and glycerol TGs [34]. PE (18:3/16:0) and PE (16:1/20:4) were identified as markers of glycerophospholipid metabolism, which may be related to the improvement in lipid droplet formation in adipose tissue and reduction in TG content. We found that TG (14:0/18:3/20:4), TG (14:0/18:4/20:3), TG (18:1/18:3/18:3), TG (14:0/20:4/22:4), TG (18:2/18:3/20:3), TG (18:2/18:3/22:5), and TG (16:0/20:4/22:6) were markers of cholesterol metabolism; TG (14:0/18:2/20:5) and TG (14:0/18:2/22:6) could be used as markers of thermogenesis; TG (14:0/18:2/18:4), TG (18:2/18:3/20:5), and TG (14:0/20:4/22:6) were found to be markers of IR; TG (14:0/16:1/18:3), TG (14:1/14:1/20:2), TG (14:1/14:1/22:3), TG (14:0/18:2/18:3), TG (18:2/18:3/18:3), TG (14:0/20:3/20:5), and TG (16:1/16:1/22:6) were found to be markers of the cAMP signal channel. In addition, there were also markers associated with fat digestion and absorption, vitamin digestion and absorption, and sphingolipid metabolism. To summarize, the anti-obesity effect of COSM may be associated with a variety of pathways, as well as the cross-talk among various pathways. However, importantly, it should be noted that most markers are associated with the cAMP signalling pathway.

AMP-activated protein kinase (AMPK) is a widely distributed serine/threonine protein kinase involved in a range of metabolic processes that play a vital role in energy metabolism and catabolism in WAT [28,35,36]. GLUT-4 is a key glucose transporter that promotes the use of extracellular glucose by insulin-sensitive cells to maintain blood glucose homeostasis [37,38]. In other words, a decrease in GLUT-4 in skeletal muscle and lipoprotein expression and the inhibition of the translocation of GLUT4 to the cell surface lead to insufficient glucose transport and hence insulin resistance [36]. Importantly, there is substantial evidence that the activation of AMPK stimulates GLUT4 translocation to the cell surface, thereby promoting glucose uptake in adipose tissue [37]. Administration of COSM significantly upregulated GLUT4 and AMPK expression. Combined with the above data for blood glucose, insulin, and HOMA-IR, our results indicate that COSM can improve glucose uptake and insulin sensitivity through the AMPK-GLUT4 pathway, thus improving IR and achieving weight loss. Interestingly, promoting glucose-stimulated insulin secretion from pancreatic β-cells can promote glucose and FFA uptake into sWAT to provide fuel for use as a substrate for UCP1 activation [39]. UCP-1 exists in mitochondria and plays a key role in mitochondrial oxidative respiration and fatty acid oxidative thermogenesis. AMPK activation and PGC-1α recruitment to the UCP1 promoter induce UCP1 expression [40]. Through mechanistic research, COSM was found to induce PGC1α and UCP1 expression by regulating AMPK to promote the burning of white fat [41]. Therefore, this mechanism may be responsible for the increased level of FFAs in the COSM group identified in the lipidomic analysis, promoting the expression of UCP1. Furthermore, AMPK is a major regulator of energy homeostasis and can regulate SREBP1, an important transcription factor in adipogenesis [41,42]. SREBP1 increases cholesterol metabolism by regulating the lipid synthesis factor SCD1 [43,44]. COSM downregulates the expression of SREBP1 and SCD1 proteins, indicating that COSM inhibits endogenous cholesterol synthesis. CPT1B is the rate-limiting enzyme that transports fatty acyl coenzymes to the inner mitochondrial membrane during fatty acid oxidation, and AMPK is the main regulator of CPT1B activity [41]. Indeed, malonyl-CoA, which is generated by ACC, is a potent inhibitor of CPT1B [41,45]. Therefore, by phosphorylating and inhibiting ACC, AMPK decreases the pool of malonyl-CoA, which results in decreased lipid synthesis and increased fatty acid import into mitochondria for β-oxidation [46]. SREBP1 is also the main regulator of fatty acid metabolism. It increases fatty acid oxidation by regulating the expression of TG and the fatty acid synthesis downstream factor ACC [47]. In summary, COSM activated the AMPK-GLUT4 pathway to regulate glucose homeostasis and improve insulin resistance. It also activated the AMPK-PGC1-UCP1 pathway to promote thermogenesis, thereby increasing fat burning. Additionally, it inhibited the de novo synthesis pathway of lipids and cholesterol by activating the AMPK-SREBP1-SCD1 pathway, reducing lipid production, and promoted the β-oxidation of mitochondria by activating the AMPK-CPT1B-ACC1 pathway. All of these results show that COSM plays an important role in the anti-obesity effect of AMPK.

## 4. Materials and Methods

### 4.1. Materials

COSM (MW ≤ 3000 Da; DD ≥ 90%; lot no.: 170311C) was purchased from Shandong AK Biotech Co., Ltd. (Qingdao, China). Table A1 provides more information on COSM. Orlistat was obtained from Zhongshan Wanhan Pharmaceutical Co., Ltd. (Guangzhou, GuangDong, China). Triglyceride (TG), total cholesterol (TC), low-density lipoprotein-cholesterol (LDL-C), high-density lipoprotein-cholesterol (HDL-C), glucose (GLU), and nonesterified fatty acid (NEFA) levels were measured using commercially available kits (Nanjing Jiancheng Bioengineering Institute, Nanjing, China). Insulin, hormone sensitive lipase (HSL), tumour necrosis factor α (TNF-α), interleukin 1β (IL-1β) and interleukin 6 (IL-6) levels were measured by ELISA kits (Mei mian, Yancheng, China).

### 4.2. Animal Experimental Design and Sample Collection

Thirty-two male C57BL/6J mice (10–15 g, 3 weeks old) were supplied by Hunan Slake Jingda Laboratory Animal Co., Ltd. (Hunan, China) and housed in a specific pathogen-free (SPF) environment (air temperature 24 ± 2 °C, relative humidity 50–60%, number of air changes > 15 times/h, 12 h light/12 h dark cycle). All procedures were conducted on the basis of the protocol (Experiment No. gdpulacSPF2017117) that was authorized by the Guangdong Pharmaceutical University Institutional Animal Care and Use Committee. After 7 days of adaptive feeding with common feed, mice were stochastically divided into a normal food (NF) group, HF group, COSM + HFD (COSM) group, and orlistat + HFD (orlistat) group (n = 8 animals per group). The normal diet (containing 20% protein, 0% carbohydrate, 4.3% fat, 4.8% cellulose, and 1.96% mineral) was provided by the Laboratory Animal Center of Guangdong Pharmaceutical University. The HFD (60% kcal fat, containing 26.2% protein, 25.6% carbohydrate, 38.7% fat, 6.0% cellulose, and 6.0% mineral) was purchased from Research Diets, USA, D12492. Table A2 provides more information on the HFD.

According to prior studies by our research group, COS was administered at high, medium, and low doses, and we found that high doses had better activity [48]. Therefore, in this study, a high dose of 1.71 g/kg/d was chosen as the administration dose. On the basis of the recommended dose for the human body and dose conversion, the oral dose of the positive control drug orlistat in mice was 45 mg/kg. To ensure that the above drug doses are precisely given to the mice, we used COSM and orlistat as the solute, and deionized water as the solvent to prepare two gavage liquids with a concentration of 171 mg/mL for the COSM group and the orlistat group. The NF and HF groups were administered normal saline. The injection volume was 0.01 mL/g of body weight. During the experiment, food and water were freely available for all mice, and we observed the appearance signs and physiological state of mice daily for abnormalities. The weight and food intake of each mouse were recorded weekly. If the body weight of the mice fed an HFD exceeded the average body weight of mice in the NF group by 20%, then the mice were considered to be obese. This calculation is called the obesity index, which is determined by the following equation: obesity index (%) = [(actual weight of HFD mice—average weight of NF mice)/average weight of NF mice] × 100% [18]. Eventually, all the mice in the high-fat diet group were successfully modelled without any deaths.

At the end of the 20-week experimental period, we collected faeces from mice in an SPF environment, and later, the mice were fasted for 12 h with access to water. Blood was collected from the mice under ether anaesthesia via retro-orbital bleeding with a capillary tube. Then, the blood was centrifuged at 4 °C at 3000 rpm for 15 min to obtain supernatant serum samples. Liver and adipose tissues (visceral adipose tissue (vWAT), subcutaneous adipose tissue (sWAT), and interscapular brown adipose tissue (BAT)) were collected. All samples were quickly frozen in liquid nitrogen and stored at −80 °C. Portions of vWAT, sWAT, and BAT were fixed in 4% paraformaldehyde for adipose histological analysis. In addition, visceral adipose tissue and subcutaneous adipose tissue are known as white adipose tissue (WAT).

The study was carried out in strict accordance with the International Guiding Principles for Biomedical Research Involving Animals and in accordance with the guidelines for ethical review of animal welfare.

### 4.3. Oral Glucose Tolerance Test (OGTT)

All mice were fasted for 12 h in advance but supplied with water. According to the body weight of the mice, a glucose solution (2.0 g/kg) was injected. The blood glucose level of each mouse was measured from the tail at 0 min, 15 min, 30 min, 60 min, 90 min, and 120 min by a Roche blood glucose metre. The area under the curve (AUC) was calculated via trapezoidal integration.

### 4.4. Biochemical Analysis

Measurement of serum indicators: The serum sample was moved from the −80℃ refrigerator to the 4℃ refrigerator, and the serum was allowed to thaw for approximately 2 h. Then, the serum sample was centrifuged at a low speed at 3000 rpm for 10 min, and the supernatant was obtained for GLU, insulin, HSL, NEFA, TC, TG, LDL-C, and HDL-C kit protocols. The homeostasis model assessment of insulin resistance (HOMA-IR) was determined using the following formula: HOMA-IR = [fasting blood glucose (mmol/L) × fasting insulin (mU/L)]/22.5 (coefficient 22.5 is the correction factor) [49,50,51].

Measurement of faecal indicators: Approximately 0.5 g of dry faeces was weighed and placed into a centrifuge tube, and 1.8 mL ether was added. After repeated shaking, the samples were placed on a shaker for 15 min. Then, the supernatant was collected, and the total cholesterol (TC) content was determined by commercial kits. For TG content in faeces, dried faeces were weighed to approximately 0.5 g, and 6 mL of 95% ethanol was added. After repeated shaking, the samples were extracted three times in a 60 °C water bath for 45 min each time, and the collected supernatants were finally combined. The combined supernatant was evaporated by heating to obtain the precipitate. Finally, 20 mL of 60% acetic acid (distilled water, *v*/*v*) was added to dissolve the precipitate, and the TG content of the faeces was determined by commercial kits.

Measurement of adipose tissue indicators: The obesity state is generally associated with low-grade chronic inflammation of adipose tissue, and it is more common in visceral adipose tissue [52,53,54]. Visceral adipose tissue was used for TNF-α, IL-1β, and IL-6 ELISA kit detection.

The above operation procedures were implemented in strict accordance with the kit instructions.

### 4.5. Adipose Pathological Analysis

Adipose tissue specimens fixed in 4% paraformaldehyde were made into paraffin sections and cut into 4 μm slices. For haematoxylin–eosin (H&E) staining, the slices were stained with H&E (Leagene Biotechnology, Beijing, China) [55].

For immunohistochemistry (IHC) experiments, sWAT tissue sections were dewaxed and hydrated with xylene and gradient ethanol. Then, the cells were permeabilized with 0.5% Triton X-100 for 10 min. The samples were blocked with goat serum for 1 h, and incubated with the primary antibody overnight at 4 °C. Then, they were incubated with the secondary antibody for 1 h at room temperature (RT) and washed with 1 × TBST. Staining was performed with a 1:1 volume of TBST and 3,3′-diaminobenzidine (DAB) diluent developer. Then, the samples were restained and mounted.

### 4.6. Lipid Profiling

Lipidomics serves as a powerful approach to illustrate the interrelation between mechanism and phenotype directly. In previous studies, Hou et al. analysed the basal level of lipid composition of two white adipose tissues from male C57BL/6J mice through targeted lipidomics and generated detailed lipid profiles. They found higher basal levels of glycerolipids in sWAT than in vWAT [53]. Therefore, in our study, lipidomics of the sWAT from the NF group, HF group, and COSM group was performed via LC–MS with established methods [56,57].

Preparation of fat samples: After thawing the adipose tissue sample, 50 mg portions were added to the corresponding 2 mL centrifuge tubes, and the weight of each sample was recorded. Then, 1 mL of lipid extract and 2 or 3 steel balls were added, and the sample was shaken well. The samples were homogenized in a ball mill for 5–7 min, and the steel balls were removed. The samples were then vortexed for 2 min and sonicated for 5 min. Next, 500 μL of deionized water was added, and the samples were vortexed for 1 min and centrifuged at 12,000 rpm at 4 °C for 10 min. Next, 500 μL of each supernatant was transferred to a 1.5 mL centrifuge tube, concentrated, lyophilized, reconstituted with 100 μL of mobile phase B, and used for LC–MS analysis.

Metabolomic conditions: Lipids were separated using a reverse-phase UPLC ACQUITY HSS T3 C18 column (2.1 mm × 100 mm × 1.8 μm, Waters, Milford, MA, USA). The analytical conditions were as follows: column temperature, 40 °C; flow rate, 0.4 mL/min; injection volume, 5 μL; solvent system, water (0.04% acetic acid); acetonitrile (0.04% acetic acid); gradient program: 95:5 *v*/*v* at 0 min, 5:95 *v*/*v* at 11.0 min, 5:95 *v*/*v* at 12.0 min, 95:5 *v*/*v* at 12.1 min, and 95:5 *v*/*v* at 14.0 min. LIT and triple-quadrupole (QQQ) scans were acquired on a triple-quadrupole-linear ion trap mass spectrometer (QTRAP) LC-MS/MS system equipped with an electrospray ionization (ESI) turbo ion-spray interface, operating in positive and negative ion mode, and controlled by Analyst 1.6.3 software (Sciex). The ESI source operation parameters were as follows: source temperature 500 °C; ion spray voltage (IS) 5500 V (positive), −4500 V (negative); the ion source gas I (GSI), gas II (GSII), and curtain gas (CUR) were set at 55, 60, and 25 psi, respectively; the collision gas (CAD) was high. Instrument tuning and mass calibration were performed with 10 and 100 μmol/L polypropylene glycol solutions in QQQ and LIT modes, respectively. A specific set of MRM transitions was monitored for each period according to the metabolites eluted within this period.

Sample quality control analysis: An equal volume solution was absorbed from each fat sample extraction solution and mixed to prepare the quality control (QC) sample. To ensure good repeatability of the analysis process, the QC sample was inserted every 10 test samples during sample injection and analysis, and the QC sample essential spectrum data was collected at least 6 times for retention time (Rt) and peak area (Area) abundance deviation, which were analysed to investigate the instrument stability.

Data processing and analysis: The MS data were preprocessed with Profinder (Agilent, Walter Brun, Germany) and then analysed with MPP software (version B.15.1, Agilent, Germany). Analyst 1.6.3 software was used to process the MS data, and PCA and OPLS-DA were used to screen the differentially expressed biomarkers, which were imported for qualitative matching with a local database (MetWare database).

### 4.7. Western Blot Assay

An amount of 200 g subcutaneous adipose tissue was added into a 1.5 mL centrifuge tube with 3 steel balls and 1 mL RIPA buffer containing protease inhibitor and phosphate inhibitor (Dalian Meilun Biotechnology Co., Ltd., Dalian, China). Subcutaneous adipose tissue samples were homogenized at 10,000 rpm for 6 min using a high-throughput tissue grinder (LAWSON SCIENTIFIC, Leighton Buzzard, UK). The lysate was then centrifuged at 12,000 rpm at 4 °C for 30 min to remove the precipitate. The protein concentration in the samples was examined using a BCA protein assay kit (Beyotime, Shanghai, China). Equal amounts of protein were separated via SDS–PAGE and transferred to PVDF membranes (Millipore, Billerica, MA, USA). 1XSDS loading buffer was added to each sample, and the buffer and protein sample were mixed in a 1:1 volume ratio. Then, these samples were placed in a 98 °C metal bath for 10 min for denaturation. A total of 40 µg of protein sample was added to each lane of the gel, and after electrophoresis, the proteins were transferred to a PVDF membrane, which was washed 3 times with 1× TBST. The membranes were then blocked with 5% nonfat milk in Tris-buffered saline with Tween (TBST) for 1 h at RT and incubated with primary antibodies overnight at 4 °C. After primary antibody incubation, the membranes were rinsed three times in TBST followed by incubation with horseradish peroxidase (HRP)-conjugated secondary antibody for 1 h at RT. The primary antibodies used included AMPK, p-AMPK, CPTIB, ACC, p-ACC, GLUT4, SREBP1, SCD1, PGC1-α, and GAPDH (Abcam plc. Shanghai, China). GAPDH was used as a loading control. The Gel DocXR+ gel imaging system (Bio–Rad Laboratories, Hercules, CA, USA) was used to image the PVDF membrane. Greyscale analysis of Western blot bands was performed with ImageJ version 1.48 (National Institutes of Health, Bethesda, MD, USA).

### 4.8. Statistical Analysis

All data are expressed as the mean ± SEM. Statistical analysis was performed via one-way ANOVA using GraphPad Prism 9.0 software (GraphPad Software, Inc., La Jolla, CA, USA). *p* value < 0.05 was considered to indicate a significant difference.

## 5. Conclusions

Overall, the present study illustrated that COSM improved glycolipid metabolism, enhanced energy expenditure and further prevented obesity in HFD-fed mice. According to lipidomics analysis, these beneficial effects relate to metabolic differences in TG, PE, DG, and SM contents in adipose tissue. Additionally, insulin resistance, thermogenesis, cholesterol metabolism, glyceride metabolism, and cyclic adenosine monophosphate (cAMP) are potential anti-obesity signalling pathways. Furthermore, we think that the AMPK pathway is an extremely valuable direction to explore the anti-obesity mechanism of COSM.

## Figures and Tables

**Table 1:** Effect of an HFD on body weight and obesity in C57BL/6J mice (n = 8, mean ± SEM).

Group	Weight before Modelling (g)	Weight after Modelling (g)	Weight after Administration (g)	Obesity Index
NF	13.2 ± 1.3	25.2 ± 4.9	27.3 ± 4.0 ***	-
HF	13.1 ± 1.2	34.0 ± 8.6 ^##^	45.9 ± 5.7	34.9%
Orlistat	14.1 ± 1.4	33.1 ± 3.3 ^##^	39.0 ± 6.3 **	31.3%
COSM	13.7 ± 2.1	32.6 ± 3.8 ^##^	38.9 ± 7.6 **	29.4%

Note: compared with the NF group, ^##^ *p* < 0.01; compared with the HF group, ** *p* < 0.01, and *** *p* < 0.001.

**Table 2:** Metabolic markers and related pathways of subcutaneous adipose tissue among the NF group, HF group and COSM group.

No.	Marker	Class	Index	Metabolic Pathway
1	DG (16:1/20:4/0:0)	Diglycerides	LIPID-P-0203	Glycerolipid metabolism
2	PE (18:3/16:0)	Phosphatidylethanolamines	LIPID-P-0600	Glycerophospholipid metabolism
3	PE (16:1/20:4)	Phosphatidylethanolamines	LIPID-P-0620	Glycerophospholipid metabolism
4	SM (d18:1/26:1)	Sphingomyelins	LIPID-P-0740	Sphingolipid metabolism
5	TG (12:0/12:0/18:2)	Triglycerides	LIPID-P-0833	Sphingolipid signalling pathway
6	TG (14:0/16:1/18:3)	Triglycerides	LIPID-P-0973	cAMP signalling pathway
7	TG (14:1/14:1/20:2)	Triglycerides	LIPID-P-0974	cAMP signalling pathway
8	TG (14:1/14:1/22:3)	Triglycerides	LIPID-P-1033	cAMP signalling pathway
9	TG (14:0/18:2/18:3)	Triglycerides	LIPID-P-1034	cAMP signalling pathway
10	TG (14:0/18:3/18:3)	Triglycerides	LIPID-P-1081	Fat digestion and absorption
11	TG (14:0/18:2/18:4)	Triglycerides	LIPID-P-1082	Insulin resistance
12	TG (14:0/18:3/20:4)	Triglycerides	LIPID-P-1119	Cholesterol metabolism
13	TG (16:1/16:1/20:5)	Triglycerides	LIPID-P-1120	Vitamin digestion and absorption
14	TG (14:0/18:2/20:5)	Triglycerides	LIPID-P-1121	Thermogenic
15	TG (14:0/18:4/20:3)	Triglycerides	LIPID-P-1122	Cholesterol metabolism
16	TG (18:1/18:3/18:3)	Triglycerides	LIPID-P-1126	Cholesterol metabolism
17	TG (18:2/18:3/18:3)	Triglycerides	LIPID-P-1152	cAMP signalling pathway
18	TG (14:0/20:3/20:5)	Triglycerides	LIPID-P-1155	cAMP signalling pathway
19	TG (16:1/16:1/22:6)	Triglycerides	LIPID-P-1156	cAMP signalling pathway
20	TG (14:0/18:2/22:6)	Triglycerides	LIPID-P-1158	Thermogenic
21	TG (14:0/20:4/22:4)	Triglycerides	LIPID-P-1160	Cholesterol metabolism
22	TG (18:2/18:3/20:3)	Triglycerides	LIPID-P-1164	Cholesterol metabolism
23	TG (18:3/18:3/18:3)	Triglycerides	LIPID-P-1175	Glycerolipid metabolism
24	TG (14:0/20:5/22:4)	Triglycerides	LIPID-P-1178	Glycerolipid metabolism
25	TG (18:2/18:3/20:4)	Triglycerides	LIPID-P-1179	Glycerolipid metabolism
26	TG (16:1/20:4/20:4)	Triglycerides	LIPID-P-1180	Sphingolipid signalling pathway
27	TG (14:0/20:3/22:6)	Triglycerides	LIPID-P-1182	Glycerolipid metabolism
28	TG (18:2/18:3/20:5)	Triglycerides	LIPID-P-1191	Insulin resistance
29	TG (14:0/20:4/22:6)	Triglycerides	LIPID-P-1193	Insulin resistance
30	TG (18:2/18:3/22:5)	Triglycerides	LIPID-P-1195	Cholesterol metabolism
31	TG (16:0/20:4/22:6)	Triglycerides	LIPID-P-1196	Cholesterol metabolism

Note: Index indicates the code name of the marker in the local database.

## Data Availability

The data presented in this study are available within the article.

## Supplementary Materials

The following supporting information can be downloaded at: https://www.mdpi.com/article/10.3390/molecules28145595/s1. Figure S1: QC sample mass spectrometry analysis of total ion flow (Abscissa: retention time; Ordinate: ion current intensity). Figure S2: Detected multimodal plot of MRM metabolites (Abscissa: retention time; Ordinate: ion current intensity). Figure S3: TIC overlay of QC sample with mass spectrometry detection (Abscissa: retention time; Ordinate: ion flow intensity).

## Appendix A

**Table A1:** Certificate analysis of the chitosan oligosaccharide.

Item	Standard	Result
Deacetylation (%)	285	90.2
Viscosity (cps, 20 °C)	<15	6.6
M.W (Da)	<5000	2600
Moisture (%)	≤10	5.3
Ash	≤1.0	0.8
Insoluble (%)	≤1.0	0.1
Pb (mg/kg)	≤2.0	0.10
As (mg/kg)	≤0.5	<0.025
Total plate count	<1000 cuf/g	<100
Colibacillus (cuf/100 g)	Negative	<30
Mould and yeast (cuf/g)	<25	<10
Particle size (mesh)	>100 mesh	100 mesh

**Table A2:** Detailed composition of the experimental diet (D12492, 60% kcal).

Class Description	Ingredients	Grams
Protein	Casein, Lactic, 30 Mesh	200.00 g
Protein	Cystine, L	3.00 g
Carbohydrate	Lodex 10	125.00 g
Carbohydrate	Sucrose, Fine Granulated	72.80 g
Fiber	Solka Floc, FCC200	50.00 g
Fat	Lard	245.00 g
Fat	Soybean Oil, USP	25.00 g
Mineral	S10026B	50.00 g
Vitamin	Choline Bitartrate	2.00 g
Vitamin	V10001C	1.00 g
Dye	Dye, Blue FD&C #1, Alum. Lake 35–42%	0.05 g
