# Supplementary material for: Anti-Obesity Effect and Mechanism of Chitooligosaccharides Were Revealed Based on Lipidomics in Diet-Induced Obese Mice
Source: Molecules. 2023 Jul 23;28(14):5595. doi: 10.3390/molecules28145595 (PMC10384603; doi:10.3390/molecules28145595)
Supplement: Supplementary file 1 [file molecules-28-05595-s001.zip › molecules-2494605-supplementary.pdf]

Supporting Material

During the experiment, we carried out quality control on the samples. The results are shown below:

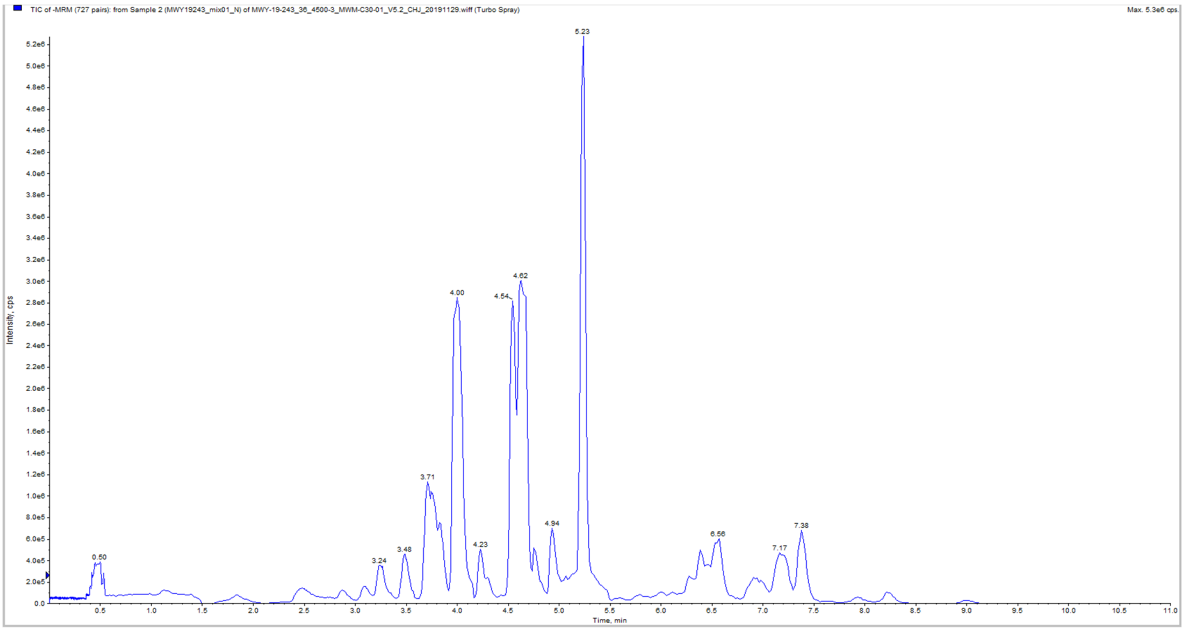

Figure S1: QC sample mass spectrometry analysis of total ion flow (Abscissa: retention time; Ordinate: ion current intensity)

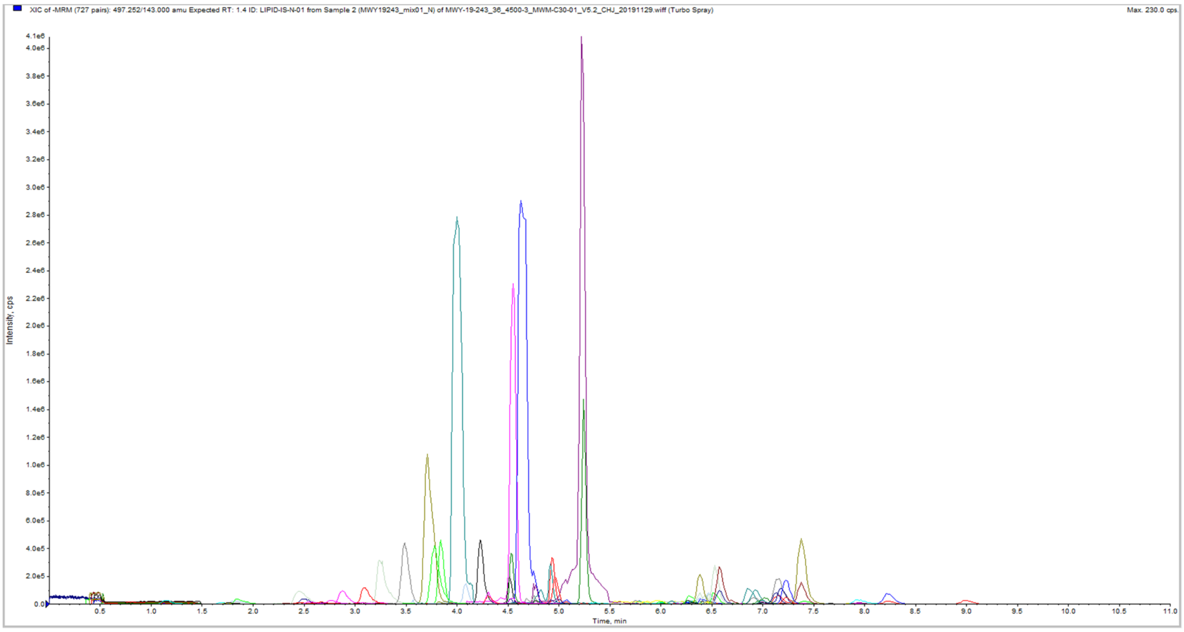

Figure S2: Detected multimodal plot of MRM metabolites (Abscissa: retention time; Ordinate: ion current intensity)

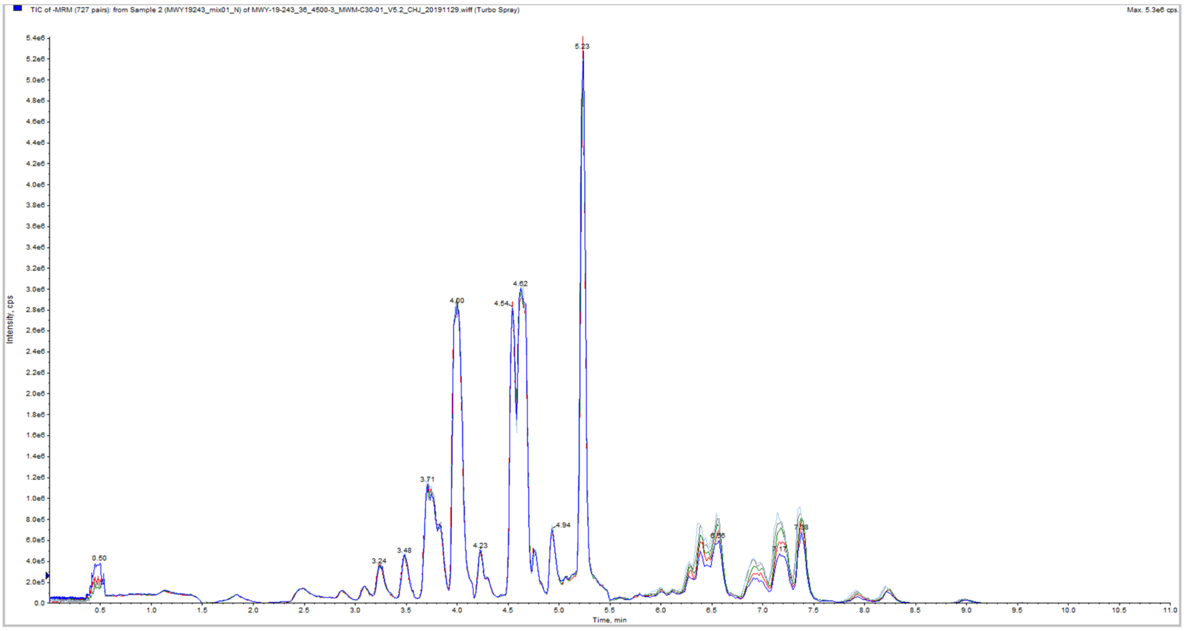

Figure S3: TIC overlay of QC sample with mass spectrometry detection (Abscissa: retention time; Ordinate: ion flow intensity)
